# Assessing the geographical distribution of comorbidity among commercially insured individuals in South Africa

**DOI:** 10.1186/s12889-020-09771-6

**Published:** 2020-11-16

**Authors:** Cristina Mannie, Hadi Kharrazi

**Affiliations:** Johns Hopkins Bloomberg School of Public Health, 25 Bowwood Road, Claremont, Cape Town, 7708 South Africa

**Keywords:** Comorbidity index, Geographical distribution, South Africa

## Abstract

**Background:**

Comorbidities are strong predictors of current and future healthcare needs and costs; however, comorbidities are not evenly distributed geographically. A growing need has emerged for comorbidity surveillance that can inform decision-making. Comorbidity-derived risk scores are increasingly being used as valuable measures of individual health to describe and explain disease burden in populations.

**Methods:**

This study assessed the geographical distribution of comorbidity and its associated financial implications among commercially insured individuals in South Africa (SA). A retrospective, cross-sectional analysis was performed comparing the geographical distribution of comorbidities for 2.6 million commercially insured individuals over 2016–2017, stratified by geographical districts in SA. We applied the Johns Hopkins ACG® System across the insurance claims data of a large health plan administrator in SA to measure comorbidity as a risk score for each individual. We aggregated individual risk scores to determine the average risk score per district, also known as the comorbidity index (CMI), to describe the overall disease burden of each district.

**Results:**

We observed consistently high CMI scores in districts of the Free State and KwaZulu-Natal provinces for all population groups before and after age adjustment. Some areas exhibited almost 30% higher healthcare utilization after age adjustment. Districts in the Northern Cape and Limpopo provinces had the lowest CMI scores with 40% lower than expected healthcare utilization in some areas after age adjustment.

**Conclusions:**

Our results show underlying disparities in CMI at national, provincial, and district levels. Use of geo-level CMI scores, along with other social data affecting health outcomes, can enable public health departments to improve the management of disease burdens locally and nationally. Our results could also improve the identification of underserved individuals, hence bridging the gap between public health and population health management efforts.

**Supplementary Information:**

**Supplementary information** accompanies this paper at 10.1186/s12889-020-09771-6.

## Background

Timely information on leading causes of mortality, disease prevalence, and health-related risk factors in various regions of a country are fundamental to setting targeted health policies. Healthcare decision makers will use such information to determine the most appropriate mix of health and social interventions to prioritize and allocate resources in each region [1, 2]. For example, community-level health information enable policymakers to address specific and changing health-related needs of individual communities in addition to the overall population [3, 4].

Considerable evidence suggests that comorbidity, in comparison to single isolated diseases, is a better predictor of current and future healthcare needs and costs [5–9]. Studies have increasingly used comorbidity-derived risk scores as valuable measures of individual health to describe and explain disease burden in populations [7, 8]. Aligning with these findings, the U.S. National Academy of Medicine recommends developing measures quantifying health status or disease burden at an individual level that can be used to better understand the distribution of risk at subnational levels as well as the overall population [10].

From a public health perspective, a comprehensive view of acute and chronic conditions impacting populations would enable a holistic assessment of health and social needs and bundling of interventions or services appropriate to best protect health [2–4]. Such a system would warrant a measure of overall health improvement while enabling new health challenges to be more easily identified, which might have been missed through approaches monitoring standalone diseases. For such a dynamic health system to exist, the timely availability and access to comprehensive health information are critical.

A few studies have aimed to describe different levels of comorbidity in South Africa [11–15]. These studies have often employed limited definitions of comorbidity, which generally focus on select chronic conditions. To our knowledge, this study provides the first large-sample, district-level description of comorbidity among commercially insured individuals in SA. Our study explores the use of a comorbidity index to provide a useful perspective on the geographical distribution of morbidity and the associated financial implications. The comorbidity index is derived from healthcare claims data that are routinely captured for commercially insured individuals. The study findings highlight a more significant role of similar population-level comorbidity studies in bridging population health management resources with national public health efforts.

## Methods

### Study objectives

The primary objectives of this study were: (1) presenting a district-level geographical distribution of the average disease burden and healthcare utilization of commercially insured individuals; (2) assessing disease burden using a measure that describes the overall disease burden of an individual (instead of isolated single diseases); and, (3) showcasing the value of health status measurement towards understanding the full picture of disease burden in SA using data that is routinely collected for healthcare delivery.

### Measure of health status

We used the validated Johns Hopkins Adjusted Clinical Grouper (ACG®) risk score as the measure of health status [16, 17]. The ACG® risk score is a patient-centred summary measure based on the premise that the sicker an individual, the more healthcare resources are required to adequately manage the individual’s health. The ACG® System classifies each individual by considering the age, gender and particular pattern of morbidity (acute and chronic conditions) experienced by the individual over the past twelve months [9]. Based on these factors as determined by available health information, each individual is assigned to one of 105 mutually exclusive ACG® risk cells. A risk score/weight is then assigned to each risk cell based on the average annual healthcare resource utilization of the individuals in each cell. Using this approach, the risk score represents the healthcare utilization expected to manage the particular combination of clinical conditions experienced by individuals in each cell [16]. The ACG® System has been shown to explain significantly more variation in utilization than demographics-only models [18, 19].

By aggregating the individual risk scores of individuals per district, the average risk score per district is determined which is known as the comorbidity index (CMI) [16]. Specific geographical areas or sub-populations identified with a CMI below or above a value of 1 are expected to experience higher or lower healthcare utilization (and implied higher or lower morbidity) compared to the average morbidity level and the average cost per life observed for the entire population. The ACG® System’s algorithms are trained using population-level U.S. claims data. We performed a standard analysis to assess the applicability of the ACG® scores within the SA context (see Additional file 1). The ACG® scores applied in this study were recalibrated using local cost data reflecting local clinical practices and benefits in SA. Age-adjusted CMIs were determined by applying the local age-specific ACG® scores to the study individuals in each age group and aggregating the individual risk scores to produce an age-adjusted CMI per district.

### Study design

A retrospective, cross-sectional analysis was performed comparing the ACG® CMI scores per district for commercially insured individuals living in the same area between 2016 and 2017. This study involved secondary data use and analysis of de-identified healthcare claims data and was approved by the Institutional Review Board at the Johns Hopkins Bloomberg School of Public Health.

### Data sources

Administrative claims data were obtained from one of the largest health risk management services providers and administrator of health plans in SA. Obtained data included: de-identified, aggregated patient-level data for 2016 and 2017 of total claims costs; the ACG® CMI risk score which provides a measure of the clinical and financial risk of each member for each year; the diagnoses and medicine categories associated with each individual; basic demographic data (age, sex, population group); and, geographical region as determined by postal codes recorded on health plan administration records.

The population groups in this study are described using the four major racial groups used by SA’s Census to classify the population [20]. Characteristics of the study population were compared to the 2011 SA Census data (provided by Statistics South Africa [20]) to measure the representation of the national population by the study denominator.

Postal codes recorded for health plan members were linked to the 2016 demarcated boundaries for electoral wards which in turn were mapped using Census data to the respective districts. Geographical shapefiles for districts in SA were obtained from publicly available sources as published by the Municipal Demarcation Board [21]. The mapping of postal codes to electoral wards and then to districts was made possible by an allocation method (developed by the claims administrator) that assigns each postal code to a mutually exclusive electoral ward.

### Study population

**Fig. 1:**
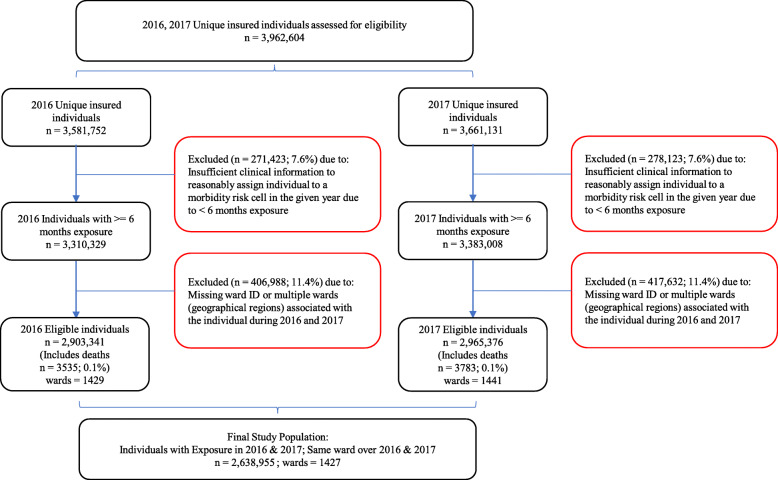
Selection process of the study population

The initial study population assessed for inclusion included 3.96 million members enrolled in SA health plans in 2016 and 2017 for whom comprehensive managed care services were provided by the administrator (Fig. 1). Health plans in SA are obligated by legislation to include benefits for a set of “Prescribed Minimum Benefits (PMBs)” which include specified emergency medical conditions, 270 specified medical events and the diagnosis, as well as treatment and management of 27 chronic conditions as stipulated in the latest “Chronic Disease List (CDL)” [22]. The study population consisted of members from open health plans (i.e., plans which anyone can join) and closed health plans (i.e., restricted to employees of organizations). All health plans were subject to conditions of open enrolment and community rating where member premiums can only be differentiated based on income and family size [23].

Two main inclusion criteria were applied to identify the final study population of 2.64 million individuals (Fig. 1). The first criterion was that members were required to have at least 6 months of membership in each year resulting in sufficient time over which to collect clinical diagnosis information for the ACG® System to reasonably assign the member to an actuarial risk cell representative of the member’s clinical and financial risk in a given year. Since individuals with less than 6 months of membership are likely to have enrolled due to a recent increased need for healthcare and therefore likely to have over-stated monthly healthcare costs, a 6-month minimum membership requirement for the assignment of ACG® risk scores is commonly recommended [16]. Hence, included individuals were identified as users and non-users of health plan benefits during the study period meeting the minimum membership requirement. Close to 83% of health plan members had 12 months membership in each year resulting in an overall average of 11.8 months each year per study life. Due to almost complete membership for the majority of the study population in each year, healthcare costs (and thus risk scores) per individual were deemed representative of annual healthcare costs.

The second criterion included members for whom a single ward (i.e. geographical location) over the two years was identified. This feature served to link the health status of members during the entire study period to a single region to which the member was exposed for an extended period likely to have impacted their concurrent health status. By ensuring the same geographical region in both years, confounding factors expected to influence healthcare utilization and the ACG® CMI risk score were assumed to be mainly constant. Figure 1 provides an annotated diagram detailing the impact of the inclusion criteria applied. It was observed that approximately 10% of members enrolled in one year were not members in the second year and vice versa due to health plan members entering and exiting health plans. Since health status is dynamic and expected to change from time to time, individuals with membership in both years were included to provide a better indication of the average morbidity level of each individual as opposed to using the morbidity level observed in only a single year.

A geo-level power and sample size analysis testing the ability to confidently detect real differences in CMI (or average healthcare utilization), when compared to the national average of at least 5%, was performed to ensure that geographical districts included in this study have adequate individuals to represent the underlying commercially insured populations in each district (see Additional file 2) [24].

### Statistical analysis

The database management and analysis for this study were performed using MySQL (v5.7.22). Statistical analysis and geographical plots were produced using R (v3.4.3). R packages used for the power analysis and geographical plots produced included pwr (v1.2–2) and tmap (v2.2) respectively. Graphs were created in Microsoft Excel 2016.

Within the ACG® System, the ACG® risk cells are used to directly explain variation in total cost and provide an easily calibrated model for explaining the relative risk [17]. The risk weights for each cell were derived by taking the mean healthcare expenditure for all individuals in a risk cell divided by the mean healthcare expenditure of all individuals in the population (indirect standardization) and scaling the means such that the average cost weight across the population is equal to one to facilitate comparison. The robustness of these risk weights when applied to different populations was tested by performing a correlation analysis between the risk weights calculated based on the ACG System’s reference U.S. population and comparing it to the risk weights calculated using local cost data for the study population. The correlation analysis showed a strong linear relationship (see Additional file 1).

## Results

### Characteristics of the study population

The 2.64 million individuals included in this study represented approximately 5% of the national population and 34% of all commercially insured members in SA [22]. In contrast to the national distribution (reported by the SA Census 2011), the study population of commercially insured individuals had a higher proportion of White members and a lower proportion of Black African members (Table 1). The commercially insured population generally consisted of older individuals (average age 31.6; SA 2017 median age 26.6) with existing healthcare needs and expected higher utilization (see Additional file 3).

**Table 1:** Population characteristics: Study population compared to South African Census 2011

	Study Population 2016 2017	South Africa Census 2011
**Unique Individuals**	2,638,955	51,764,899
Wards	1427	4277
Districts	52	52
Provinces	9	9
**Sex**		
Female	1,459,269 (55.3%)	26,579,527 (51.3%)
**Population Groups**		
Black African	1,743,515 (66.1%)	40,996,454 (79.2%)
Indian/Asian	102,229 (3.9%)	1,286,789 (2.5%)
Coloured	203,298 (7.7%)	4,614,896 (8.9%)
White	530,274 (20.1%)	4,586,336 (8.9%)
Unknown	59,639 (2.3%)	280,423 (0.5%)
**Age Bands**		
00–04	251,838 (9.5%)	5,684,973 (11.0%)
05–09	277,237 (10.5%)	4,819,353 (9.3%)
10–14	257,808 (9.8%)	4,594,492 (8.9%)
15–19	245,690 (9.3%)	5,003,087 (9.7%)
20–24	94,063 (3.6%)	5,374,063 (10.4%)
25–29	112,642 (4.3%)	5,058,738 (9.8%)
30–34	180,821 (6.9%)	4,028,532 (7.8%)
35–39	186,664 (7.1%)	3,467,343 (6.7%)
40–44	205,362 (7.8%)	2,948,218 (5.7%)
45–49	209,260 (7.9%)	2,619,908 (5.1%)
50–54	192,386 (7.3%)	2,217,920 (4.3%)
55–59	152,690 (5.8%)	1,797,131 (3.5%)
60–64	99,006 (3.8%)	1,385,535 (2.7%)
65–69	66,348 (2.5%)	957,668 (1.9%)
70–74	46,011 (1.7%)	748,204 (1.4%)
75–79	30,855 (1.2%)	481,216 (0.9%)
80–84	17,575 (0.7%)	322,870 (0.6%)
85+	12,699 (0.5%)	255,648 (0.5%)
**Province**		
Eastern Cape	292,154 (11.1%)	6,560,024 (12.7%)
Free State	221,538 (8.4%)	2,745,290 (5.3%)
Gauteng	557,680 (21.1%)	12,271,736 (23.7%)
KwaZulu-Natal	458,618 (17.4%)	10,266,802 (19.8%)
Limpopo	260,984 (9.9%)	5,404,032 (10.4%)
Mpumalanga	252,359 (9.6%)	4,039,488 (7.8%)
North West	194,919 (7.4%)	3,509,672 (6.8%)
Northern Cape	83,159 (3.2%)	1,145,529 (2.2%)
Western Cape	317,544 (12.0%)	5,822,326 (11.2%)
**Exposure**		
Average membership months per year	11.8	–
**Morbidity status**		
ACG risk score / Comorbidity index (CMI)	0.998	–

We also compared study population characteristics to the national distribution data by each of the 9 provinces in SA (Table 1). *P*-values testing for significant differences between the distribution of lives across the study population compared to the 2011 Census proved to be of little use due to the large samples in each subgroup making even small differences in proportions appearing to be significant [25, 26].

SA’s 9 provinces contain 52 districts. Districts are divided into 4277 smaller geographical units known as electoral wards [20]. The study population resided in a third of all wards (*n* = 1427) representing all 52 districts.

### Geographical distribution of study lives versus 2011 census lives

**Fig. 2:**
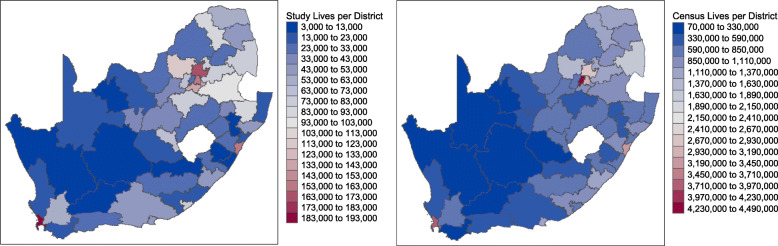
Commercially insured individuals (study population) per district (left) compared to Census lives per district (right) (Source: Author’s work)

We compared the geographical distribution of the analyzed study population to the distribution of the 2011 Census lives by district in SA (Fig. 2). The study population was concentrated in the City of Cape Town in the Western Cape (along the West coast), the City of Tshwane in Gauteng (North) and Ethekwini in KwaZulu-Natal (East coast). These cities represent three of the five most populous districts in SA. The City of Johannesburg which is the most populous city in SA was underrepresented in this study. A listing of district names per province including key variables associated with each district compiled during this study is provided (see Additional file 4). A map of labelled SA district codes is also provided as reference (see Additional file 5). The map of SA was produced in R using geographical shapefiles for districts in SA obtained from publicly available sources as published by the Municipal Demarcation Board [21].

### Geographical distribution of CMI

**Fig. 3:**
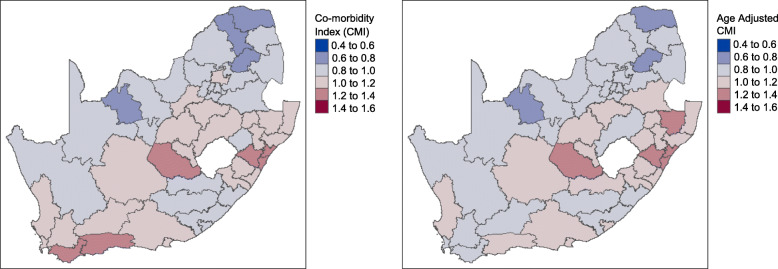
A comparison of CMI per district before (left) and after (right) adjusting for age (Source: Author’s work)

Districts within the Western Cape, Free State, and Kwa-Zulu Natal showed higher levels of disease burden (i.e., CMI values represented by shades of red) compared to other parts of the country (Fig. 3). In contrast, the Eastern Cape, and especially the Northern Cape and Limpopo provinces appeared to have the lowest CMI scores (i.e., shades of blue) in the country (Fig. 3).

Figure 3 highlights the districts in which the highest disease burden is expected based on healthcare utilization as represented by the CMI value. To interpret the CMI, each category shown in the legend represents the expected average annual healthcare cost of individuals in each district relative to the overall average healthcare cost per life of the study population. For example, the highest CMI range of 1.4 to 1.6 indicates that individuals in these districts on average are expected to incur annual healthcare costs 40 to 60% higher than the overall average cost per life of the study population. On the other end of the scale, a CMI of .4 to .6 indicates districts in which the average annual healthcare cost of individuals is expected to be 40–60% lower than the overall average cost per life of the study population. Since the CMI describes health status as a measure of healthcare utilization assumed to be based on healthcare need, areas in which access to healthcare treatment may be poorer than other areas may falsely appear to have a lower burden of disease. By classifying districts using CMI, beneficial insights into areas with highest to lowest healthcare utilization relative to each other warranting further investigation as to the causes of increased need, real relatively lower need or even potential unmet healthcare need are possible.

To better understand whether the CMI can be explained by other factors such as increased age for whom increased healthcare costs are expected, we compared changes in CMI before and after accounting for the influence of age on the individuals in each district (Fig. 3 left vs. right). A few districts in the Western Cape (e.g. Overberg and Eden) appeared to have lower CMIs after adjusting for age suggesting that some of the healthcare utilization in these areas can be explained by a higher proportion of older adults in the region (average age ≈ 40). In other areas, such as the Capricorn district in the Limpopo province (North), a higher CMI is observed after accounting for age (average age ≈ 29). Districts in the Free State and KwaZulu-Natal (districts on the left and right respectively surrounding the enclaved country of Lesotho (area in white) within the border of SA) were found to have high CMIs even after adjusting for the influence of age (Fig. 3 left and right).

### Geographical distribution of CMI by population group

**Fig. 4:**
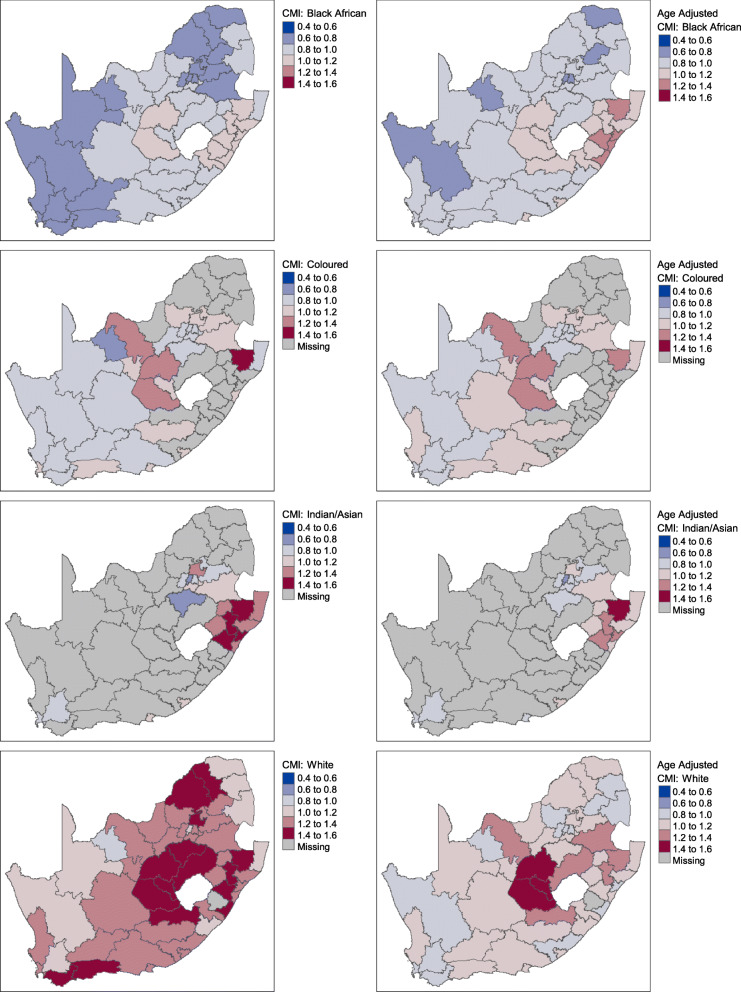
A comparison of CMI stratified by population group before (left) and after (right) adjusting for age (Source: Author’s work)

We also analyzed the influence of population grouping. Figure 4 provides a comparison of the CMI per district stratified by each population group (rows) and before and after adjusting for age (left and right columns). The CMI associated with White individuals in the study population stands out from the other population groups (i.e., geographies with shades of red in the last row of maps). Constituting only 20% of the individuals in this study, this population group is expected to incur higher than average healthcare utilization compared to other population groups. After adjusting for age, highest CMIs persist for the White population group although it is noted that the observed differences are reduced (Fig. 4). In contrast, the geographical plot of Black African individuals indicates several districts expecting to incur lower than average healthcare utilization relative to other population groups.

CMIs consistently appeared high amongst the districts within the Free State and KwaZulu-Natal provinces (surrounding Lesotho) for all population groups before and after adjusting for age (Fig. 4). This effect suggests that the high CMI (i.e., higher than average healthcare utilization due to disease burden) for individuals in these districts compared to other districts cannot be explained by age alone, and that additional factors may influence the results.

## Discussion

Comorbidities are essential predictors of cost and utilization in healthcare [8]. Prior studies have shown the geographical imbalances of individual diseases; however, assessing the geographical distribution of comorbidities, represented as a risk index of increased healthcare utilization, has not been accomplished for SA. This study examined the geographical distribution of morbidity and its associated financial implications among commercially insured individuals in SA. The study results identified geographies at risk of increased healthcare utilization and potential worsening outcomes that need the attention of both population and public health efforts.

Population analysis of individual-level risk, as presented in this study, are important sources of information for the implementation of priority public health interventions as each perspective of risk (individual and population levels) is influenced by the other [27]. The results of this study show how the disease burden of individuals aggregated at a district level can assist in identifying underlying causes of disease or dominant risk factors that may be contributing to the health status of specific groups of individuals or entire population groups. The ability to rank the risk of each individual in a population relative to each other has both retrospective and prospective applications, and has been used to support health systems with matters of finance, administration, care delivery and evaluative research [16].

Provincial-level analyses are critical for establishing overall measures of health and disease frequency across the country. They are arguably the most practical to perform since most of the national budget in SA is distributed at the provincial level [28]. The challenge, however, is that the information needed to inform ways in which the health status of a province may be improved lies at understanding the health status at lower geographical levels such as districts or even electoral wards making up the provinces and how each contribute specifically to the overall health of the SA population. It is at these levels that local sociocultural and environmental realities present and healthcare disparities can more easily be recognized and addressed [12, 27].

The use of a composite measure to assess the health status of populations is especially useful to compare the health of various areas relative to each other, identify vulnerable or disadvantaged groups, and assist in the prioritization of interventions and equitable allocation of resources [1]. In this study, districts in the Free State and KwaZulu-Natal provinces appear to have higher than expected healthcare utilization for all population groups after adjusting for age suggesting that research into other factors influencing health care utilization in these areas is required. Although the measure presented in this study represents an overall comorbidity level of individuals and not the isolated burden due to the prevalence of specific diseases, the areas detected appear consistent with other literature findings using various other definitions of disease burden [12, 15]. A superior benefit of the data presented in this study, however, is that with similar routinely captured data future analyses to track morbidity trends can be performed easier and more cost-effectively.

Study findings also showed that the Black African population group has generally lower than expected healthcare utilization relative to the White population group. Inequities in access to healthcare in SA largely due to historic legacy, the urban-rural divide and disproportionate concentration of general practitioners and specialists in private practices in urban areas are expected to play a role [22]. Indeed, differences may exist between population groups pertaining to when, how and the choice of healthcare services sought, which may produce different utilization patterns among individuals of the same disease burden [27].

The CMI as a measure of disease burden has a weakness in that it assumes that all else being equal, sicker individuals will utilize more healthcare services, when in fact the opposite may also be true (i.e. the sickest individuals may tend to use less due to lack of access to care and/or poorer longevity). While the significant geographical variation in health status across districts observed in this study may not infer a similar trend in the SA population, this study provides valuable insights regarding the distribution of individual risk amongst commercially insured individuals since the risk of the study individuals in each geographical area cannot be separated from the disease risk of the population groups to which the individuals belong [10].

Data generated in this study has significant potential to contribute towards greater public health efforts at a national, provincial, district and even electoral ward level especially if linked to national data sources such as Census data. By incorporating in risk stratification analyses social factors known to play a significant role in the health of individuals throughout life and therefore the overall health of populations, a deeper understanding of the financial impact of local determinants of health may be possible [29].

Modern-day healthcare systems will rely significantly on the discipline of health information technology to play an important role in population-level healthcare [27, 30, 31]. Population health management as a field has contributed significantly to the effective and efficient use of clinical and financial resources to manage the health of defined populations within healthcare budgets. While this is critical to the management and sustainability of health systems, current strategies are severely limited in their ability to reduce the growing number of individuals that will inevitably require higher healthcare resources unless the underlying health factors into which we are born, live and work can effectively and collectively be improved [32].

### Limitations

The results of this study should be interpreted within the boundaries of the following limitations: First, the postal code, used to assign patients to different wards and districts, was assumed to be a reliable account of latest address due to the standard capture and routine confirmation performed of personal information on record. Missing postal codes or having multiple address locations recorded may have resulted in unrepresented individuals causing some geographical bias in the study population [33]. An assessment of the completeness of geocoding and the impact of excluding such individuals from the analysis was not possible without additional information that could be used as a proxy for member location.

Second, while this study was performed on a considerably large population of 2.6 million individuals, they tend to be mostly employed in comparison with the average national unemployment rate of 27.1% for the study period [34]. Evidence suggests that “unemployed people may be more likely than employed people to visit physicians, take medications or be admitted to general hospitals” [35]. Since the study population are mostly employed and therefore likely to be healthier (due to better socio-economic circumstances in general) and incur lower healthcare costs than those unemployed, the results of this analysis may be understated particularly in geographical areas impacted by higher unemployment rates.

Third and lastly, to protect the anonymity of the members and health plans included in this study, details identifying the health plans by name or specific benefit packages offered by the health plans were not provided. Consequently, it was not possible to assess associations between specific benefit package designs and healthcare utilization (i.e., CMI). However, the legislated implementation of a prescribed minimum benefit package in SA ensures a standard level of treatment (which health plans in the private sector are obligated to fund) thus lessening some of the disparity associated with affordability and access to healthcare.

## Conclusions

A combined approach involving the targeting of high-risk populations (which tend to make up a small proportion of the population) with a greater public health approach focused on the entire population for whom prevention and primary care is required, can potentially have a higher impact on the overall burden of diseases than each approach separately [10, 36]. In addition to the prevalence of specific diseases on which reports are usually based, a view of multimorbidity can be a vital predictor of current and future healthcare spending to inform issues of resource allocation and service delivery [2]. Thus, geographically stratified CMIs can help both health plans and public health agencies to align efforts in reducing anticipated high rates of healthcare utilization while reducing disparities (e.g., access to care) on a population level simultaneously.

## Supplementary Information

**Additional file 1.** Assessing Applicability of the ACG® Score for South African Claims Data. Figure that illustrates the correlation between ACG® System weights and empirically derived weights using South African claims data.

**Additional file 2.** Power and Sample Size Analysis. Table that illustrates the power and sample size analysis.

**Additional file 3.** Characteristics of the Study Population. Figures that describe characteristics of the study population.

**Additional file 4.** List of districts per province with key indicators. Table that illustrates the districts per province and key study indicators.

**Additional file 5.** District boundaries, South Africa. Map that illustrates the district boundaries in South Africa and district codes per province.

## Data Availability

The data that support the findings of this study are available from Medscheme Pty (Ltd) South Africa, but restrictions apply to the availability of these data, which were used under license for the current study, and so are not publicly available. Data are however available from the authors upon reasonable request and with permission of Medscheme Pty (Ltd) South Africa.

## Abbreviations

SA: South Africa
ACG®: Adjusted Clinical Grouper
CMI: Comorbidity index
PMBs: Prescribed Minimum Benefits
CDL: Chronic Disease List

## References

[1] Morrow RH, Bryant JH. Health policy approaches to measuring and valuing human life: conceptual and ethical issues. Am J Public Health. 1995;85(10):1356–60. 10.2105/ajph.85.10.1356. 10.2105/ajph.85.10.1356 PMC1615602 7573617

[2] Hyder, A.A., Puvunachandra, P., & Morrow, R. (2012) In Merson MH, Black RE, Mills AJ. Global health : diseases, programs, systems, and policies. Measures of health and disease in populations. In: 3. ed. Sudbury Mass.: Jones & Bartlett Learning; 2012:936.

[3] Gamache R, Kharrazi H, Weiner JP (2018). Public and population health informatics: the bridging of big data to benefit communities. Yearbook Med Informat.

[4] Kharrazi H, Weiner J (2014). IT-enabled Community Health Interventions: Challenges, Opportunities, and Future Directions. eGEMs.

[5] Barnett K, Mercer SW, Norbury M, Watt G, Wyke S, Guthrie B (2012). Epidemiology of multimorbidity and implications for health care , research , and medical education : a cross-sectional study. Lancet.

[6] Glynn LG, Valderas JM, Healy P (2011). The prevalence of multimorbidity in primary care and its effect on health care utilization and cost. Fam Pract.

[7] De Andrade V, Crozet C, Lombrail P, Gagnayre R, Lefe T (2014). What do we mean by multimorbidity ? An analysis of the literature on multimorbidity measures , associated factors , and impact on health services organization. Revue d’Epidemiologie et de Santé Publique.

[8] Starfield B, Kinder K (2011). Multimorbidity and its measurement. Health Policy.

[9] Johns Hopkins Center for Population Health Information Technology (CPHIT) (2018). The Johns Hopkins ACG ® System White Paper.

[10] Institute of Medicine (U.S.). Committee on Assuring the Health of the Public in the 21st Century. The future of the public’s health in the 21st century. Understanding population health and its determinants. Washington, D.C.: National Academies Press; 2002. p. 509.

[11] Oni T, Youngblood E, Boulle A, McGrath N, Wilkinson RJ, Levitt NS. Patterns of HIV, TB, and non-communicable disease multi-morbidity in peri-urban South Africa- a cross sectional study. BMC Infect Dis. 2015. 10.1186/s12879-015-0750-1. 10.1186/s12879-015-0750-1 PMC4300166 25595711

[12] Weimann A, Dai D, Oni T (2016). A cross-sectional and spatial analysis of the prevalence of multimorbidity and its association with socioeconomic disadvantage in South Africa: a comparison between 2008 and 2012. Soc Sci Med.

[13] Folb N, Timmerman V, Levitt NS (2015). Multimorbidity, control and treatment of non- communicable diseases among primary healthcare attenders in the Western cape, South Africa. South Afr Med J.

[14] Ataguba J. Inequalities in multimorbidity in South Africa. Int J Equity Health. 2013;12:64. 10.1186/1475-9276-12-64 PMC3765402 23962076

[15] Wyk VP, Msemburi W, Laubscher R (2012). Mortality trends and differentials in South Africa from 1997 to 2012 : second National Burden of Disease Study. Lancet Glob Health.

[16] Johns Hopkins Bloomberg School of Public Health (2014). The Johns Hopkins ACG ® System Version 11.0 Applications Guide.

[17] Johns Hopkins Bloomberg School of Public Health. The Johns Hopkins ACG ® System Version 11.0 Technical Reference Guide. Baltimore: The Johns Hopkins University; 2014.

[18] Corti MC, Avossa F, Schievano E (2018). A case-mix classification system for explaining healthcare costs using administrative data in Italy. Eur J Int Med.

[19] Kharrazi H, Chi W, Chang HY (2017). Comparing population-based risk-stratification model performance using demographic, diagnosis and medication data extracted from outpatient electronic health records versus administrative claims. Med Care.

[20] Statistics South Africa | The South Africa I Know, The Home I Understand. http://www.statssa.gov.za/. Accessed April 17, 2019.

[21] 2016 Boundaries – District Municipalities – Municipal Demarcation Board. http://www.demarcation.org.za/site/documents/2016-boundaries-district-municipalities/. Accessed April 17, 2019.

[22] Council for Medical Schemes (2018). Annual Report 2017|2018 A Healthy Industry for All.

[23] Republic of South Africa (1998). Medical Schemes Act 131 of 1998.

[24] Health budget 2020–21 - vulekamali. https://vulekamali.gov.za/2020-21/national/departments/health/. Accessed March 22, 2020.

[25] Sullivan GM, Feinn R (2012). Using effect size—or why the P value is not enough. J Graduate Med Educ.

[26] Lin M, Lucas HC, Shmueli G (2013). Too big to fail: large samples and the p-value problem. Inf Syst Res.

[27] Gibbons MC. M.C. Gibbons MD, MPH Johns Hopkins Urban Health Institute. In: Bos L et al., Medical and Care Compunetics 5*.* Populomics*.* Amsterdam: IOS Press; 2008. p. 265–8.

[28] UNICEF (2018). UNICEF South Africa 2018 Health Budget Brief.

[29] Hatef E, Kharrazi H, Nelson K (2019). The association between neighborhood socioeconomic and housing characteristics with hospitalization: results of a National Study of veterans. J Am Board Fam Med.

[30] Dixon BE, Kharrazi H, Lehmann HP (2015). Public Health and Epidemiology Informatics: Recent Research and Trends in the United States. Yearbook Med Informat.

[31] South African National Department of Health (2019). National Digital Health Strategy for South Africa. 2019–2024.

[32] World Health Organization. About social determinants of health: WHO; 2017. https://www.who.int/social_determinants/sdh_definition/en/. Accessed April 17, 2019.

[33] Oliver N, Matthews KA, Siadaty M, Hauck FR, Pickle LW. Geographic bias related to geocoding in epidemiologic studies. Int J Health Geographics. 2005;4(29). 10.1186/1476-072X-4-29. 10.1186/1476-072X-4-29 PMC1298322 16281976

[34] Statistics South Africa (2018). P0211 - Quarterly Labour Force Survey (QLFS), 4th Quarter 2017.

[35] Jin RL, Shah CP, Svoboda TJ (1997). The impact of unemployment on health: a review of the evidence. J Public Health Policy.

[36] Frieden TR (2010). A framework for public health action : the health impact pyramid. Am J Public Health.

