# Supplementary material for: Assessing the geographical distribution of comorbidity among commercially insured individuals in South Africa
Source: BMC Public Health. 2020 Nov 16;20:1709. doi: 10.1186/s12889-020-09771-6 (PMC7667849; doi:10.1186/s12889-020-09771-6)
Supplement: Supplementary file 1 — Additional file 1. Assessing Applicability of the ACG® Score for South African Claims Data. Figure that illustrates the correlation between ACG® System weights and empirically derived weights using South African claims data. [file 12889_2020_9771_MOESM1_ESM.docx]

**Additional file 1: Assessing Applicability of ACG^®^ Score for South African Claims Data**

The ACG® System uses clinical diagnoses captured by insurance claims to assign individuals to the most appropriate risk cell representative of the individuals’ clinical and financial risk. A limitation of this method is that a condition identified by diagnosis codes of an individual in a given year which continues to persist but is not specifically recorded in the subsequent years may result in a different ACG® morbidity risk cell assignment with fewer conditions reported to explain the clinical risk associated with the resource utilization observed. To ensure a more comprehensive view of an individual’s true level of morbidity, the average of the morbidity risk score in 2016 and 2017 was used to represent the overall morbidity level of each health plan member.

The weights of ACG® risk cells are based on healthcare claims costs derived for a defined U.S. reference population at a specific point in time (i.e., base year). The weights assigned to each risk cell describe the average expected cost of lives in that cell relative to other ACG® risk cells. Within the same ACG® risk cell, individuals may have different combinations of clinical conditions with similar resource utilization. Users of the ACG® System often compare the weights provided by the system to those derived using healthcare costs for individuals where possible, to assess the appropriateness of the standard U.S. weights generated by the ACG® System within the local context.

A strong positive correlation (Pearson correlation coefficient = 0.99) between the ACG® weights based on the U.S. reference population and the ACG® weights empirically derived using the local SA population was observed (Figure A1). Since either of the two sets of weights could have reasonably been considered for use in this study, ACG® users are encouraged to calculate risk scores using local cost data if available “to more accurately reflect local benefits and area practice patterns”^[[1]](#footnote-1)^. Based on these results we decided to use the empirically derived weights to account for any local differences that exist (i.e., we retrained the ACG® algorithm to generate new weights for each of the predictors).

Figure A1 Correlation between ACG® System weights and empirically derived ACG weights used in this study

Weights for each ACG® risk cell were based on 2016 healthcare costs. The relative weights determined for each ACG® cell were then applied to the individuals assigned to the same ACG® risk cell in 2017.

1. Johns Hopkins Bloomberg School of Public Health. *The Johns Hopkins ACG ® System*

   *Version 11.0 Applications Guide*.; 2014. [↑](#footnote-ref-1)
