# Supplementary material for: Assessing the geographical distribution of comorbidity among commercially insured individuals in South Africa
Source: BMC Public Health. 2020 Nov 16;20:1709. doi: 10.1186/s12889-020-09771-6 (PMC7667849; doi:10.1186/s12889-020-09771-6)
Supplement: Supplementary file 2 — Additional file 2. Power and Sample Size Analysis. Table that illustrates the power and sample size analysis. [file 12889_2020_9771_MOESM2_ESM.docx]

**Additional file 2: Power and Sample Size Analysis**

Since healthcare needs can vary from individual to individual, it is expected that measures based on healthcare costs too may vary considerably from individual to individual. After applying the ACG® CMI risk score to the study population, the standard deviation of 1.6313 was calculated which showed a wide spread of individual risk scores below and above the overall population average risk score of 0.9982. To assess the number of individuals within each geographical area required to detect notable differences in CMI with a reasonably high certainty, a power analysis was performed using a two-sided t-test. A dearth of literature pertaining to the minimum detectable difference in CMI considered significant lead to various scenarios describing minimum detectable differences in CMI ranging from 5% to 30% at 80% and 90% power respectively being tested (Table B1).

Initially, the geographical unit considered was electoral ward, but this resulted in only a small proportion of wards having sufficient individuals to detect smaller differences in CMI with an acceptable level of certainty, for example, only 5.5% of all wards –accounting for 44.2% of the study population– had enough individuals to detect a statistically significant difference (alpha=0.05) in CMI of 5% with 80% certainty. At a district level, however, almost all districts contained sufficient individuals to enable the detection of significant small differences in CMI (i.e., as small as 0.05) with 80% and 90% certainty. Thus, districts were chosen as the unit of geographical analysis for this study.

Table B1 Power and sample size analysis

| Power | Minimum detectable difference in CMI | Sample size required per geographical unit | Number of wards with individuals >= sample size (%) | Number of individuals in wards >= sample size (%) | Number of districts with individuals >= sample size (%) | Number of individuals in districts >= sample size (%) |
| --- | --- | --- | --- | --- | --- | --- |
| 0.8 | 0.05 | 8381 | 78 (5.5%) | 1,166,502 (44.2%) | 48 (92.3%) | 2,614,522 (99.1%) |
| 0.8 | 0.10 | 2090 | 310 (21.7%) | 2,140,117 (81.1%) | 52 (100%) | 2,638,955 (100%) |
| 0.8 | 0.20 | 522 | 657 (46.0%) | 2,523,794 (95.6%) | 52 (100%) | 2,638,955 (100%) |
| 0.8 | 0.30 | 232 | 864 (60.5%) | 2,598,532 (98.5%) | 52 (100%) | 2,638,955 (100%) |
| 0.9 | 0.05 | 11,232 | 46 (3.2%) | 855,390 (32.4%) | 47 (90.4%) | 2,603,706 (98.7%) |
| 0.9 | 0.10 | 2799 | 249 (17.4%) | 1,989,929 (75.4%) | 52 (100%) | 2,638,955 (100%) |
| 0.9 | 0.20 | 699 | 585 (41.0%) | 2,480,622 (94.0%) | 52 (100%) | 2,638,955 (100%) |
| 0.9 | 0.30 | 311 | 791 (55.4%) | 2,578,833 (97.7%) | 52 (100%) | 2,638,955 (100%) |
