# Supplementary material for: Assessing the geographical distribution of comorbidity among commercially insured individuals in South Africa
Source: BMC Public Health. 2020 Nov 16;20:1709. doi: 10.1186/s12889-020-09771-6 (PMC7667849; doi:10.1186/s12889-020-09771-6)
Supplement: Supplementary file 3 — Additional file 3. Characteristics of the Study Population. Figures that describe characteristics of the study population. [file 12889_2020_9771_MOESM3_ESM.docx]

**Additional file 3: Characteristics of the Study Population**

A typical characteristic of the SA commercially insured population is the severe under-representation of healthy individuals (who opt-out in early adulthood due to the absence of mandatory membership) coupled with an over-representation of older individuals. Figure C1 highlights this phenomenon which is evident by the markedly fewer individuals observed amongst those aged 20-29 years and the higher proportion of individuals over 40 years compared to the national distribution. Since a high proportion of members over 40 years (compared to younger individuals) will tend to have increased healthcare needs, the lack of young, healthier members on health plans restricts the ability of health plans to be able to subsidize older, sicker members in a community-rated environment resulting in a need for higher premiums. This, in turn, reduces the affordability of private healthcare for the majority of the population.^[[1]](#footnote-1)^

Figure C1 Comparing the distribution of the study population versus South African total population by age band

Women tend to be higher users of healthcare than men.^[[2]](#footnote-2)^^[[3]](#footnote-3)^ Some of the reasons cited for this general global phenomenon include different health-seeking behaviour observed amongst men and women (e.g. women tend to report signs and symptoms earlier than men would, as well as the natural biological and gynaecological events in the life-course of women). Figure C2 illustrates this same tendency within the study population of women to consume more healthcare services than men as illustrated by a higher CMI during their life course from the ages of 15-64 years. However, from the age of 64 years, men can be seen to experience a steeper increase in healthcare utilization due to increased healthcare needs surpassing women of the same age.

Figure C2 CMI by sex and age band

Figure C3 illustrates the proportion of individuals per age band and in comparison, with the corresponding proportion of healthcare expenditure incurred by each age band. While individuals over 60 years of age make up only 10% of the population, they account for approximately a third of all costs. In comparison, children under 19 years of age makeup 39% of the population but account for only 14% of total healthcare expenditure.

Figure C3 Percentage of total healthcare expenditure by age band among the SA study population

Understanding population dynamics such as these can assist not only with adequate concurrent and future healthcare resource planning (e.g. anticipated healthcare needs based on the age, sex and life expectancy of the population) but importantly also with the prioritization of the most appropriate mix of effective multi-sectoral measures aimed at creating by default, social, living and working conditions protective of health throughout one’s life course.^[[4]](#footnote-4)^^[[5]](#footnote-5)^ To illustrate the extent to which the study population is representative of the national distribution at a district level, Figure C4 provides a distribution of the commercially insured population across all 52 districts relative to the SA population. It also indicates the proportion of the SA population included in the study to highlight specific districts in which greater or poorer representation exists which should be considered when interpreting results. For example, the figure shows that the districts of Fezile Dabi in the Free State (province) and Sedibeng in Gauteng (province) have the highest percentages (15.7% and 15.5% respectively) of individuals from those areas represented in the study population. On the other hand, the City of Johannesburg, one of the most populous metropoles in Gauteng and the country, is severely under-represented in our study with only 0.6% of Census 2011 individuals reported included for analysis.

Figure C4 A comparison of the distribution of commercially insured individuals represented in this study with the distribution of 2011 Census populations per district

1. Council for Medical Schemes. *Annual Report 2017|2018 A Healthy Industry for All*.; 2018. [↑](#footnote-ref-1)
2. Carretero MT, Calderón-larrañaga A, Poblador-plou B, Prados-torres A. Primary health care use from the perspective of gender and morbidity burden. *BMC Women’s Health*. 2014;14:145. [↑](#footnote-ref-2)
3. Redondo-sendino Á, Guallar-castillón P, Banegas JR, et al. Gender differences in the utilization of health-care services among the older adult population of Spain. *BMC Public Health*. 2006;6(1):1-9. doi:10.1186/1471-2458-6-155 [↑](#footnote-ref-3)
4. Morrow RH, Bryant JH. Public Health Policy Forum Health Policy Approaches to Measuring and Valuing Human Life: Conceptual and Ethical Issues. *American Journal of Public Health*. 1995;85(10) [↑](#footnote-ref-4)
5. Redondo-sendino Á, Guallar-castillón P, Banegas JR, et al. Gender differences in the utilization of health-care services among the older adult population of Spain. *BMC Public Health*. 2006;6(1):1-9. doi:10.1186/1471-2458-6-155 [↑](#footnote-ref-5)
