# Supplementary material for: Assessing the geographical distribution of comorbidity among commercially insured individuals in South Africa
Source: BMC Public Health. 2020 Nov 16;20:1709. doi: 10.1186/s12889-020-09771-6 (PMC7667849; doi:10.1186/s12889-020-09771-6)
Supplement: Supplementary file 4 — Additional file 4. List of districts per province with key indicators. Table that illustrates the districts per province and key study indicators. [file 12889_2020_9771_MOESM4_ESM.docx]

**Additional file 4: List of districts per province with key indicators**

Table D1 List of districts per province with key study indicators
