# Supplementary material for: Assessing the geographical distribution of comorbidity among commercially insured individuals in South Africa
Source: BMC Public Health. 2020 Nov 16;20:1709. doi: 10.1186/s12889-020-09771-6 (PMC7667849; doi:10.1186/s12889-020-09771-6)
Supplement: Supplementary file 5 — Additional file 5. District boundaries, South Africa. Map that illustrates the district boundaries in South Africa and district codes per province. [file 12889_2020_9771_MOESM5_ESM.docx]

**Additional file 5: District boundaries, South Africa**

**
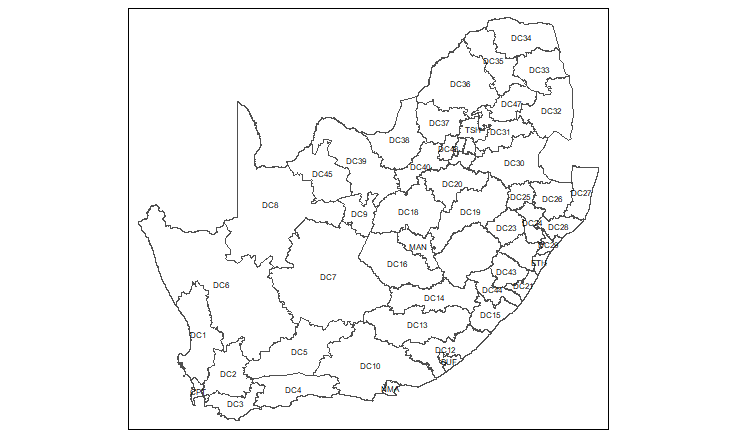
**

Figure E1 District boundaries, South Africa

*All 52 district names can be found listed in numerical order according to district code (by province) in Additional file 4.
